# The London School of Tropical Medicine

**Published:** 1905-05-06

**Authors:** 


### THE LONDON SCHOOL OF TROPICAL MEDICINE.

The third list of stewards which has just been issued in connection with the festival banquet at the Hotel Cecil on the 10th instant, at which the Right Hon. Joseph Chamberlain, M.P., will preside, reveals the deep interest taken in this admirable school by the leaders of public opinion in all ranks and professions. It is likely to be a memorable gathering full of interest from many points of view, not the least of which is the important work which the London School of Tropical Medicine has already accomplished for humanity during the relatively few years of its existence. The school in the Victoria and Albert Docks is admirably equipped, and the students show a tendency to increase in importance and number each year. The hospital in connection with the school contains some of the most interesting and best planned and equipped hospital wards in the world, and nothing that foresight and brains could accomplish has been left undone to make this school worthy in all respects of the metropolis of the Empire. We will not anticipate the proceedings on the 10th instant by indicating the considerable achievements of the members of this school, but will content ourselves with wishing Mr. Chamberlain a bumper collection—for of course money is seriously needed—and the dinner committee such a success that the banquet may rank for all time as memorable in the annals of charity. The sincere acknowledgments of all will be gladly given to the chairman of the dinner committee, the Duke of Marlborough, and his colleagues, to the honorary secretaries, Mr. William Waldorf Astor and Sir Ralph Moor, and to Mr. P. J. Michelli, the indefatigable secretary, to whom so much genuine credit for the success already achieved is in reality due.

